# Clinical Trial Design and Regulatory Requirements for Artificial Intelligence as a Medical Device: A PRISMA-ScR–Guided Scoping Review of Global Guidance and Evidence (2017–2025)

**DOI:** 10.3390/jcm15051937

**Published:** 2026-03-04

**Authors:** Umamaheswari Shanmugam, Mohan Kumar Rajendran, Jawahar Natarajan, Veera Venkata Satyanarayana Reddy Karri

**Affiliations:** 1 Department of Pharmaceutics, JSS College of Pharmacy, JSS Academy of Higher Education & Research, Nilgiris, Ooty 643001, Tamil Nadu, India; uma.maye@gmail.com (U.S.);; 2 Magnaait Ltd., London WC2H 9JQ, UK

**Keywords:** AI as a medical device, SaMD, scoping review, clinical trial design, regulatory science, adaptive validation

## Abstract

**Background**: Artificial Intelligence as a Medical Device (AIaMD) introduces regulatory, methodological, ethical, and clinical challenges that are not fully addressed by traditional device trial frameworks. Given rapidly evolving and jurisdiction-specific guidance, a consolidated mapping of trial design expectations and regulatory requirements is needed. **Objective**: To map regulatory requirements and clinical trial design approaches for AIaMD across major jurisdictions and to identify key methodological and implementation gaps relevant to adaptive/continuously learning systems. **Methods**: A scoping review was conducted in accordance with the PRISMA-ScR reporting guideline. Peer-reviewed literature (2017–2025) was searched in PubMed, Embase, Web of Science, and the Cochrane Library. Gray literature was identified from major regulators and policy bodies (FDA, EMA, MHRA, PMDA, WHO, CDSCO). Eligible records addressed AIaMD clinical evaluation, trial design, regulatory pathways, post-market surveillance, or reporting standards. Data were charted using a predefined extraction framework and synthesized descriptively with thematic analysis across regulatory, methodological, ethical, and clinical implementation domains. **Results**: Included sources demonstrate substantial heterogeneity in evidence expectations and AI-specific pathways across jurisdictions. Recurrent themes include the need for predefined change management, performance monitoring and drift controls, dataset representativeness and bias evaluation, transparency and versioning, cybersecurity, and real-world evidence integration. Reporting frameworks (SPIRIT-AI, CONSORT-AI, MI-CLAIM) are frequently cited as mechanisms to improve reproducibility and regulatory readiness. **Conclusions**: Evidence and regulatory expectations for AIaMD remain fragmented. Harmonization of terminology, trial design principles, and post-market governance—supported by standardized reporting—would improve clinical validity, safety assurance, and scalability across regions. This review has several limitations. As a scoping synthesis, it prioritizes breadth of coverage rather than quantitative meta-analysis. Included sources vary in methodological rigor and reporting detail, and evolving regulatory guidance may change rapidly over time. Nevertheless, integrating peer-reviewed and regulatory evidence provides a comprehensive overview of current expectations and emerging gaps. In conclusion, effective evaluation of AIaMD requires a shift from static, one-time validation toward continuous lifecycle oversight that integrates adaptive trial designs, transparent reporting standards, bias surveillance, and structured post-market monitoring. Regulatory heterogeneity currently poses significant barriers to multinational development; however, coordinated adoption of standardized evidence frameworks and collaborative governance mechanisms may reduce duplication while preserving patient safety. By translating methodological principles into operational guidance, this review aims to support regulators, sponsors, and clinical investigators in designing trials that are both scientifically rigorous and practically implementable for continuously learning systems.

## 1. Introduction

### 1.1. Definition of AI as a Medical Device (AIaMD) & Software as a Medical Device (SaMD)

The field of medicine has benefited tremendously through AI: Improvement in diagnosis, treatment planning, patient experience, and outcomes; however, its operation in healthcare requires regulatory frameworks [1]. When AI systems fulfill the criteria of a medical device for regulatory authorities (such as the United States Food and Drug Administration-FDA and the European Medicines Agency-EMA), they are called AI as a Medical Device (AIaMD). On the other hand, Software as a Medical Device (SaMD) is software meant to be used for medical purposes whose functions may include clinical decision support or diagnostic system-a function; however, that need not necessarily require the presence of a hardware system [2,3]. This poses both a benefit and an obstacle for regulation because AI technologies and SaMD technologies are usually caught in the gray areas between traditional software and regulated medical devices, calling for a hybrid wear-and-tear regulatory framework combined with AI-relevant guidelines.

Unlike existing descriptive summaries of AI medical device regulation, this scoping review critically compares regulatory approaches across jurisdictions, identifies key methodological and evidentiary gaps in AIaMD clinical validation, and proposes a practical, lifecycle-oriented roadmap for harmonized trial design, reporting, and post-market governance. The objective is to provide actionable guidance for regulators, sponsors, and investigators developing adaptive AI systems.

### 1.2. Role of Clinical Trials in AI Device Validation

Prior to safe and effective usage in clinics, AI-based devices have to undergo rigorous clinical validations. Clinical trials serve as the gold standard, validating the safety and efficacy of medical devices, thus guaranteeing their intended functioning in the real world [4]. AI-oriented clinical trials look at issues related to the intended clinical outcome of the device but also endeavor to deal with pertinent issues such as model training, data quality, and generalizability. Since AI systems are rather continuously learning systems, the trial design or methodology has to cater for this iterative improvement, something that is foreign to the traditional paradigms for drug or medical device testing [5]. AI trials will also require shifting the evaluation of performance from and alongside measures like accuracy, sensitivity, specificity, and patient safety, including long-term data tracking, to ensure that the device continues to perform satisfactorily with the new data being fed as it learns [6].

### 1.3. Regulatory Complexity and the Global Push for Harmonization

The regulatory scene of AI medical devices is, indeed, highly complex as it lies at the intersection of existing medical device laws and emerging AI regulations. Different organizations in different parts of the world have handled the risks and opportunities that AI brings to healthcare in different ways. For instance, the FDA has come up with a Software Precertification Program, while the EMA has given guidelines for SaMDs along the framework of Medical Device Regulation (MDR). There is no world consensus on evaluating AI, whereby standards in aspects of data quality, transparency, and approval processes differ [7]. Such regulatory fragmentation may affect AI devices’ development and distribution on a cross-border scale, thereby hindering market access and limiting the scalability of AI technology in healthcare [8]. Lately, there has been a loud call, spearheaded by regulatory agencies and academia alike, to harmonize AI medical device standards globally so as to ease adoption and ensure patient safety and product efficacy [1].

### 1.4. Research Gap and Objectives of the Review

Despite rapid growth in AIaMD regulatory guidance and clinical evaluation literature, evidence expectations remain heterogeneous across jurisdictions and clinical domains, with inconsistent terminology, variable requirements for adaptive or continuously learning systems, and fragmented recommendations on trial design, reporting, and lifecycle governance.

A scoping review methodology is appropriate to map the breadth of regulatory and methodological sources, identify recurring themes and gaps, and compare how evidence expectations differ across regions.

The objectives of this scoping review were:

(i) to map regulatory requirements relevant to AIaMD clinical evaluation across major jurisdictions;
(ii) to summarize clinical trial design approaches proposed or used for AIaMD; and
(iii) to identify methodological, reporting, and implementation gaps affecting safe and scalable deployment.

### 1.5. Clinical and Assistive Practice Applications

Artificial Intelligence as a Medical Device (AIaMD) is increasingly introduced into clinical pathways as a decision-support or assistive technology rather than as an autonomous replacement for clinician judgment. Evidence suggests that the clinical value of AIaMD is most likely to be realized when systems are integrated within established workflows, accompanied by clearly defined human oversight, accountability structures, and safety controls [5,9].

From an implementation perspective, AIaMD may support earlier detection of deterioration, improved risk stratification, and more efficient allocation of specialist resources in high-burden settings. For example, AI-assisted prescreening tools have demonstrated potential to improve patient identification and recruitment efficiency in oncology trials [10]. Similarly, AI-enabled teledermatology services have shown feasibility and diagnostic performance in real-world practice environments [11], and imaging or cardiovascular decision-support systems may assist clinicians with prioritization of high-risk cases [5].

However, translation from development environments to routine practice introduces risks that may not be fully captured during controlled trials. Model performance may degrade over time when exposed to heterogeneous sites or evolving data sources, underscoring the importance of post-market surveillance and lifecycle monitoring [7,12]. Algorithmic bias has also been reported to affect performance across demographic subgroups, potentially amplifying inequities if not proactively assessed and mitigated [13,14]. Furthermore, usability, interpretability, and human–AI interaction factors substantially influence safe adoption and clinician trust [5,9].

For these reasons, clinical readiness requires evaluation beyond accuracy metrics alone. In addition to diagnostic performance, studies increasingly recommend assessing workflow integration, usability, safety monitoring, and real-world effectiveness to determine whether AIaMD can be safely adopted at scale [9,15].

### 1.6. International Landscape

The global landscape for regulating AI as a medical device (AIaMD) remains highly fragmented, and a number of regulatory agencies have established distinct ways of doing it, with the FDA initiating a regulatory framework for AI devices in the US, including its own Software Precertification Program. The program enables software developers to prove their own capacity for managing AI that continues to evolve after-the-market, with the key focus being on safety and effectiveness [1]. Whereas in Europe, AI medical devices are governed under Medical Device Regulations (MDR) as a subcategory of SaMD. They aim to ensure safety, performance, and traceability of AI devices [16]. It is important to note that, similarly, the Japanese Pharmaceuticals and Medical Devices Agency (PMDA) regulates AI devices and issues guidelines harmonizing very closely with the MDR, yet having certain localized variances in data requirements and clinical validation procedures [4].

In the UK, the MHRA has been involved in the development of guidelines specifically tailored for AI, aiming at ensuring the clinical safety of AI systems and thus promoting a culture of innovation. Unlike the FDA, the MHRA places importance upon harmonization with European regulations, owing to its historical incorporation of EU frameworks [17]. The CDSCO in India has recently stepped up its efforts to regulate AI-driven medical devices, though regulatory processes are still evolving. The World Health Organization (WHO) has called for global standards in AI regulation, recognizing the increasing integration of AI in healthcare and the need for international regulatory cooperation [17]. These various regulatory bodies present challenges, not only for developers but also for healthcare providers seeking to adopt AI systems across borders.

### 1.7. Differences Between Medical Device Laws and AI-Specific Regulation

One primary challenge in regulatory mechanisms posed is the overlap between traditional laws for medical devices and those considered for AI systems. Medical device laws have traditionally been grounded in the physical hardware of a fixed set of unchanging functionalities [1]. Contrarily, AI systems evolve with processing new data, thereby creating a paradox where applying existing static regulation against such dynamic systems proves difficult. Post-market learning and updating of the AI devices stands in stark contrast to traditional laws: it demands a novel regulatory approach. The FDA, for example, has proposed a set of guidelines focused on the continuous learning paradigm of AI, where real-world evidence and post-market monitoring are critical components of the validation process [4]. This is quite the opposite of the traditional model, whereby clinical trials are held before approval.

However, fewer and farther between are the mandated options available for AI, a fact whose very flexibility engenders potential concerns over ensuring safety and efficacy over time. Among these concerns, for instance, under the FDA premarket requirement for AI devices, proving initial safety and performance is an essential concept; however, post-market monitoring of said devices, and their learning capability on a continuous basis, must also be performed, which is somewhat different from processes used for traditional medical devices [5]. The global regulatory drive has created varied approaches to control for innovation with issues of patient safety; thus, harmonized regulation must be set up.

### 1.8. Post-Market Surveillance Requirements

Post-market surveillance is crucial, however, to ensure the safety and effectiveness of AI medical devices once they have been launched. Unlike traditional medical devices that require rigorous clinical trials prior to their approval for marketing, AI devices may need continuous monitoring due to their learning abilities. The FDA emphasizes the continuing evaluation through the Software Precertification Program that allows AI systems to be updated and tracked during their lifecycle [7]. The reliance on real-world data and continuous monitoring is a fundamental distinction in regulating AI devices, which learn and evolve through use.

The Member States of the European Union have put the MDR into force, mandating continuous post-market surveillance to ensure that AI devices continue to meet safety standards as they interact with new patient data [8]. Both the MHRA and CDSCO have adopted PMA as part of their regulatory processes for AI device regulation, but both jurisdictions face the challenge of aligning these processes with international standards. In India, post-market surveillance jurisprudence is still in its infancy, with rudimentary guidelines focusing on clinical data after launch but lacking a concrete mechanism for ongoing updates [1].

The WHO has also advocated for international standards on the regulation of AI medical devices, underlining the importance of ensuring post-market surveillance globally [18]. As more AI medical devices enter healthcare systems around the world, international consistency in post-market surveillance would be crucial for ensuring patient safety and improving the efficiency of regulatory activities.

## 2. Methods

### 2.1. Study Design

This study was conducted as a scoping review to map and synthesize regulatory guidance, methodological literature, and clinical evidence relating to Artificial Intelligence as a Medical Device (AIaMD). A scoping approach was selected due to the heterogeneous, interdisciplinary, and rapidly evolving nature of AIaMD research, where study designs, regulatory frameworks, and outcomes vary substantially, making quantitative synthesis or meta-analysis inappropriate.

The review aimed to identify key themes, regulatory expectations, trial design adaptations, and implementation considerations rather than to estimate effect sizes or formally compare interventions. Reporting follows the PRISMA-ScR guideline to enhance transparency and reproducibility.

A protocol defining objectives, eligibility criteria, and data-charting fields was developed prior to screening. Given the exploratory scope and inclusion of regulatory gray literature, the protocol was not prospectively registered.

Quality appraisal and reporting alignment:

Consistent with scoping review methodology, sources were not excluded based on methodological quality. However, to enhance transparency, Joanna Briggs Institute (JBI) appraisal tools were applied as an informative assessment of reporting and methodological limitations according to source type. Reporting conforms to PRISMA-ScR, and the checklist and flow diagram are provided (Supplementary Materials; flow diagram also included in the main manuscript).

Instruments, devices, and software:

Reference management was performed using EndNote 21 (Clarivate, Philadelphia, PA, USA). Study screening (title/abstract and full-text eligibility assessment) was conducted using Rayyan (Qatar Computing Research Institute, Doha, Qatar; web application; https://rayyan.ai; accessed on 31 March 2025). Data charting and manuscript preparation were performed using Microsoft Excel and Microsoft Word, version 365 (Microsoft Corp., Redmond, WA, USA). No specialized analytical or statistical software was used.

### 2.2. Information Sources and Search Strategy

A structured literature search was conducted across PubMed, Embase, Web of Science, and the Cochrane Library to identify relevant publications from January 2017 to March 2025. This timeframe was selected to capture the period during which formal AI-specific regulatory guidance and clinical trial methodologies began to emerge.

Search terms combined controlled vocabulary and free-text keywords using Boolean operators, including:

“Artificial Intelligence as a Medical Device” OR “AIaMD” OR “Software as a Medical Device” AND “clinical trial” OR “clinical validation” OR “regulatory framework” OR “post-market surveillance”.

To ensure regulatory completeness and real-world applicability, gray literature was additionally identified through official publications and guidance documents from major regulatory and policy authorities, including the U.S. Food and Drug Administration (FDA), European Medicines Agency (EMA), Medicines and Healthcare products Regulatory Agency (MHRA), Pharmaceuticals and Medical Devices Agency (PMDA), World Health Organization (WHO), and the Central Drugs Standard Control Organization (CDCSO, India).

The full electronic search strategy for PubMed is provided in the Supplementary Materials.

### 2.3. Eligibility Criteria

Sources were selected according to predefined eligibility criteria.

Inclusion criteria:

(i) Focus on artificial intelligence or machine-learning systems classified as medical devices or Software as a Medical Device (SaMD).
(ii) Discussion of clinical trial design, clinical validation, regulatory approval pathways, or post-market surveillance.
(iii) Peer-reviewed research articles, regulatory guidance, or authoritative policy documents.
(iv) English language publications.

Exclusion criteria:

(i) Non-medical AI applications.
(ii) Purely technical or algorithm-development studies without a clinical or regulatory context.
(iii) Editorials, opinion pieces, or commentaries lacking methodological or regulatory relevance.

### 2.4. Selection of Sources of Evidence

**Figure 1:**
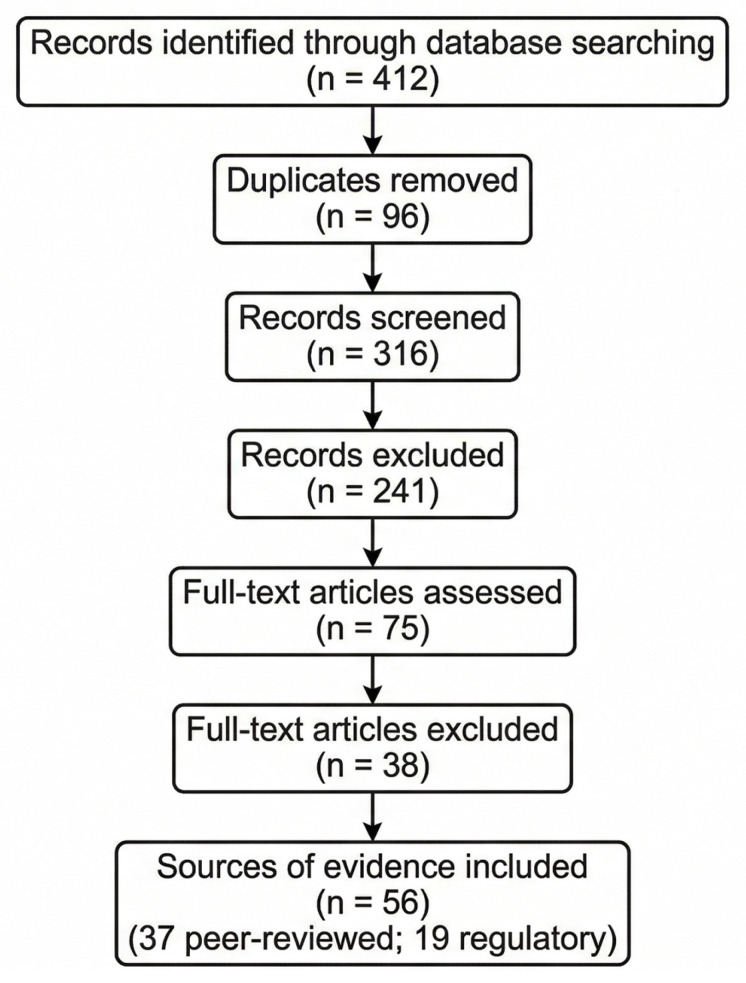
PRISMA-ScR flow diagram of the study selection process.

Titles and abstracts were screened for relevance, followed by full-text assessment against eligibility criteria. Records were excluded when they did not meet the inclusion criteria. The study selection process is summarized in the PRISMA-ScR flow diagram (Figure 1).

### 2.5. Data Charting and Synthesis

Data from included sources were charted using a standardized extraction framework capturing:

(i) Jurisdiction or regulatory body.
(ii) Device category or clinical domain.
(iii) Clinical trial design characteristics.
(iv) Validation and performance metrics.
(v) Lifecycle management and post-market surveillance requirements.
(vi) Reporting or documentation standards.

Extracted information was synthesized descriptively using thematic analysis to identify recurring regulatory, methodological, ethical, and clinical implementation patterns across jurisdictions. Findings are presented narratively and comparatively rather than quantitatively.

### 2.6. Quality Considerations

Consistent with scoping review methodology, the objective of this study was to map the range and nature of available evidence rather than to formally exclude studies based on methodological quality. Regulatory guidance and peer-reviewed sources were prioritized, and limitations of individual publications are acknowledged where relevant in the narrative synthesis.

### 2.7. Study Selection Results

The search yielded 412 records across databases. After removal of 96 duplicates, 316 records underwent title and abstract screening, of which 241 were excluded. Seventy-five full-text articles were assessed for eligibility, with 38 excluded due to insufficient clinical or regulatory relevance.

A total of 37 peer-reviewed publications were included. Additionally, 19 regulatory or policy documents from major international authorities (FDA, EMA, MHRA, PMDA, WHO, CDSCO) were included as gray literature.

In total, 56 sources of evidence were synthesized.

## 3. Clinical Trial Design Adaptations for AI

### 3.1. Trial Phases Tailored for AI

An assortment of clinical trial designs is purposely fashioned to bridge the phase-wise architecture traditionally used in medicine with the unique nature of AI when speaking of its development and evaluation as a medical device. In typical trials for medical devices, the phase before the pivotal RCTs entails pre-clinical feasibility studies, and there are post-marketing surveillance stages [5]. With regard to AI systems, feasibility studies may also constitute a stage comprising simulation-based evaluations with pre-recorded retrospective datasets ahead of the evaluation through clinical testing [2,3].

Table 1 illustrates the differences in trial phases for traditional devices versus AIaMD. Notably, AI trials often involve iterative updates and revalidation even during the pivotal phase, as algorithmic performance can be sensitive to new data sources [17].

### 3.2. Adaptive Designs and Continuous Learning Systems

One of the traits of AI is its ability to learn continuously, which restricts standard RCT designs. The so-called adaptive design regulates its operations in an attempt to monitor its progress and therefore offers a fitting trial setting for interventions with artificial intelligence [19]. Algorithms, for example, can be re-trained partway through trials to remove any biases present or integrate new imaging modalities, though with guards against overfitting and inadvertent performance drift [13].

The matter of actual ethical oversight becomes higher with adaptive trials of AI when an update to the algorithm may place patient safety at risk without the algorithm’s being validated yet [6]. The MI-CLAIM checklist [12] provides a means of ensuring transparency in updates of the model, data sources, and validation metrics throughout adaptive trials.

**Figure 2:**
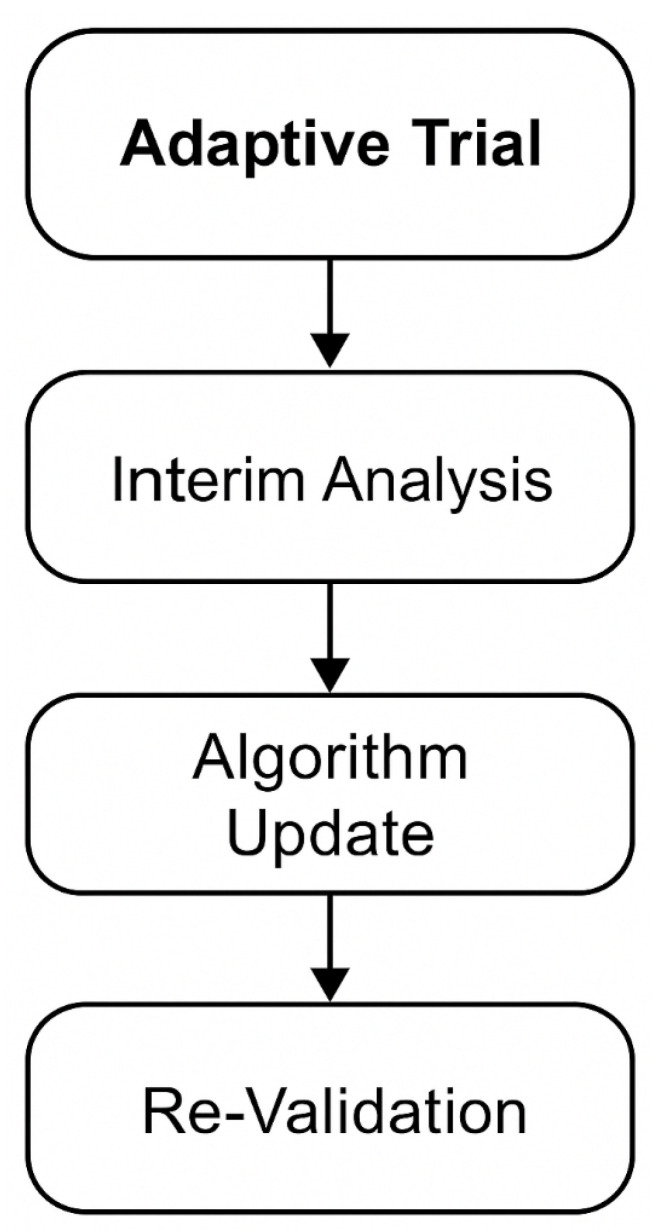
Adaptive trial cycle for AIaMD, integrating interim analyses, algorithm updates, and re-validation phase.

Figure 2 illustrates an adaptive trial cycle for AIaMD, integrating interim analyses, algorithm updates, and re-validation phases (adapted from Lee and Lee, 2020 [19]; Norgeot et al., 2020 [12]).

### 3.3. RCTs Versus Real-World Evidence (RWE)

Although random clinical trials still represent the validation process with regard to efficacy and safety, the ever-changing nature of AI models and their dependency on real-world data point toward the importance of RWE [14]. Refs. [2,3] suggest that hybrid designs mixing study components from RCTs and RWE capture performance in uncontrolled settings all over with varying specificity and hence have better generalizability.

Ref. [15] provides empirical evidence that insurance claims-based RWE can capture AI device adoption trends and highlight disparities in performance across patient demographics, thus complementing RCTs by finding biases or safety issues that occur after-market.

Mello-Thoms et al. [20] identify RWE as capable of showing benefits relative to operations and workflow in medical imaging that traditional efficacy RCTs cannot, whereas RWE must be collected in a manner that is harmonized to allow comparisons and minimize confounding.

### 3.4. Role of Digital Twins and Simulation in Pre-Clinical Testing

Digital twins, digital versions of a patient or any clinical system, are now fundamental for the development of algorithms in the pre-clinical AI testing stage (2019). Such simulations provide the perfect environment to stress-test algorithms in different situations before moving into patient-facing trials.

Ref. [21] asserts that for AI-driven imaging systems, simulation trials can swiftly ascertain the performance of devices across a broad spectrum of disease states without the logistical constraints of accessing a patient cohort diversely constituted of age, sex, and perhaps medical history.

Simulation environments add another ethical sustenance to the reduction in exposure of patients to unproven algorithms for treatment [6]. It is particularly critical for high-risk interventions such as oncology or cardiovascular treatments, where an erroneous decision of the algorithm can translate into tragic consequences (Table 2).

## 4. Data Compliance

### 4.1. Dataset Representativeness and Annotation Quality

The base of any artificial intelligence as a medical device (AIaMD) is its development and validation through datasets of good quality and representativeness. Dataset representativeness ensures that the AI works within its anticipated patient population, clinical setting, or disease prevalence rate [22]. Datasets lacking in representativeness risk generating a model that tends to perform well in lab conditions but poorly outside in real-world situations, especially across diverse demographic and geographic situations [13]. This problem is accentuated in the case of AIaMD, where diagnoses and treatment decisions make a great impact on patient outcomes. The care with annotation is equally important. Poorly annotated datasets introduce label noise, which could propagate errors through the entire model development cycle. Ref. [22] emphasizes that annotation processes must be standardized and, if possible, more than one expert rater should be involved and consensus protocols should be followed to minimize inter-observer variability. The SPIRIT-AI guidelines also require open disclosure of characteristics of the studied datasets, including annotation protocols, to enable reproduction and appraisal by regulators [9] (Table 3).

### 4.2. Bias Detection and Mitigation Strategies

Bias in AI models is one of the key issues, because it may result in systematic and ingrained disparities in healthcare delivery. Bias can occur if certain subgroups of patients are underrepresented in training data sets, if class distributions are not balanced, or simply because data may have socio-economic and geospatial limitations [13,14]. Where AIaMDs are involved, these biased algorithms can magnify health inequalities, lessen confidence in healthcare, and imperil patient safety. Therefore, in order for this to be avoided, the pre-deployment validation should have included stratified analyses for performance across pertinent subpopulations. Ref. [14] contends for a multidisciplinary approach to mitigating bias, including statistical rebalancing techniques, focused data augmentation, and socio-ethical supervision during design. Post-market surveillance may also be engaged in identifying new biases when the model is confronted with novel populations [7]. The MI-CLAIM checklist reiterates the need to document bias detection methods in the name of transparency and accountability [12].

### 4.3. Privacy Compliance (HIPAA, GDPR)

Data sharing and privacy issues pose a legal and ethical concern to AIaMD developers. In the United States, HIPAA acts as a principal law governing data sharing and storage, while the GDPR differs in Europe by imposing stricter consent and data minimization requirements. The distributed approach proposed by [8] opens avenues to work around existing privacy constraints by enabling federated learning models, so that patient data resides within local healthcare institutions, with model parameters updated from a central authority.

While compliance is paramount, establishing trust follows closely. As such, any ethical AI trial would take codesign approaches toward privacy, including encryption, secure data transfer, and anonymization from the outset of model development [6]. With the data flowing across country borders, we would require harmonization of HIPAA, GDPR, and other regional laws (e.g., India’s Digital Personal Data Protection Act) to enable multinational AIaMD trials, without compromising compliance.

**Figure 3:**
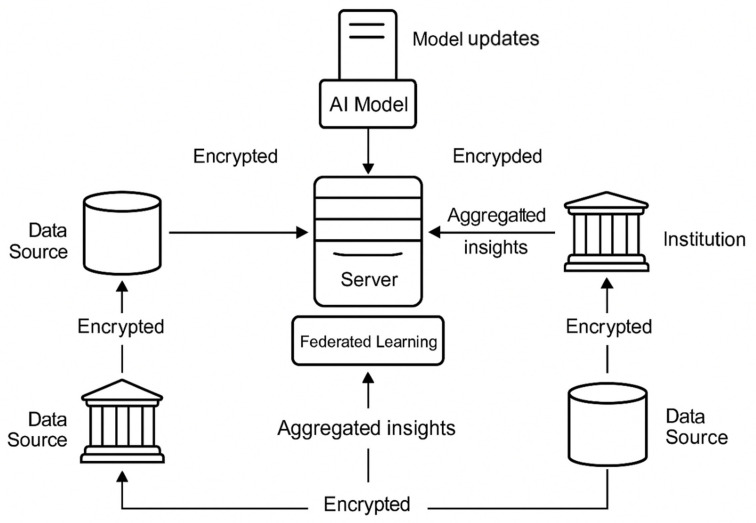
Schematic overview of privacy-preserving AIaMD development workflows.

Figure 3 provides a schematic overview of privacy-preserving AIaMD development workflows, integrating federated learning and encryption protocols.

## 5. Ethical and Legal Considerations

### 5.1. Informed Consent in AI Trials

Informed consent is a basis of ethical clinical research, yet AI-powered medical devices pose a peculiar challenge in communicating their function, limitations, and potential risks to participants. Unlike traditional medical devices, AI systems, whatever their name, interdisciplinary tech-those using machine learning are often operating under the aura of a “black box,” leaving the patient unable to fully perceive the decision-maker. According to [6], for informed consent to be valid in AI trials, participants should be made aware of the data used, potential algorithmic updates to the procedure, and how the performance of the AI itself may vary over time. In essence, researchers need to extend beyond the usual consent form and start to employ a dynamic consent process that changes as the AI systems themselves go through an evolution throughout the trial lifecycle.

Another factor that aggravates this challenge is digital literacy varying from person to person, thereby necessitating personalized educational material [14]. If this is not done, then chances are the participant will agree to consent without ever actually comprehending, which might render the whole trial ethically tainted.

### 5.2. Transparency, Accountability, and Explainability

Transparency is described as a property of an AI clinical trial entailing algorithmic and procedural openness. This stretches from the sharing of the raw data and architectures of models to the validation treatment. Ref. [9] in the SPIRIT-AI Extension has maintained that protocols on trials for AI intervention must include clear standards for reporting to allow reproducibility and trustworthiness.

Responsibility remains quite understandably at the forefront when AI outputs go directly into clinical decisions. Ref. [13] indicates that bias within the training data sets may lead to dangerous situations unless these are corrected, thus obliging an explicit delineation of responsibility amongst developers, clinicians, and regulators alike. Also, explainability-the ability to interpret and understand how decisions are imposed by an AI-is paramount to the trust of clinicians and acceptance of patients. This would be of high importance in areas such as oncology and cardiology, wherein high-risk algorithmic output would influence life-saving interventions [5].

### 5.3. Ethical Deployment and Patient Safety

Ethical implementation of AI medical devices during trials must first protect the patient in a controlled setting and even thereafter in a real-world circumstance. According to [6], there should be checks to detect algorithmic drift before preventive methods are applied. It may be observed that the performance of AI worsens with time as the input distributions change. This is more important in adaptive AI systems, which update themselves after deployment (Table 4).

Safety also concerns cybersecurity, as [18] indicates: if compromised, AI systems could harm a patient if attackers appropriately manipulate the decision outputs. Regulatory bodies are guiding the integration of security risk assessments into trial design, so such threats are anticipated.

### 5.4. Comparative Global Privacy Considerations in AIaMD Trials

While HIPAA primarily governs protected health information within U.S. healthcare entities, GDPR imposes stricter requirements on consent, data minimization, cross-border data transfer, and financial penalties for non-compliance. These differences have significant implications for multinational AIaMD trials, particularly those involving centralized data processing or real-time algorithm updates.

Emerging regulations, such as India’s Digital Personal Data Protection Act, introduce additional localization and consent requirements, further complicating cross-border AI clinical research. To address these challenges, AIaMD trials increasingly adopt privacy-preserving strategies such as federated learning, decentralized data storage, and early-stage data flow mapping aligned with the strictest applicable regulatory framework.

## 6. Performance Validation and Reporting Standards

### 6.1. Key Performance Metrics in AI Clinical Trials

Measurement of diagnostic and prognostic accuracies is essential for the performance evaluation of AI medical devices. Accuracy, sensitivity, specificity, and the area under the receiver operating characteristic curve (ROC-AUC) are the measures of choice for determining not only if the system works as supposed to but also whether its performance meets or surpasses that of human beings [11]. For example, sensitivity is required to ensure true positive cases are intercepted efficiently in screening settings like cancer detection; specificity aids in the reduction of false positives, which can lead to unnecessary interventions [16].

Relying on just one metric may provide misleading results, especially for highly imbalanced datasets. Hence, it is important to consider more metrics and stratify results across different subgroups, including demographic variables, to ensure fair performance [23].

#### Operationalizing Dynamic Informed Consent in AIaMD Trials

To move beyond conceptual discussion, dynamic informed consent in AIaMD trials can be implemented using a tiered framework. Entry-level consent may include written explanations supplemented by short educational videos. Advanced-level consent may incorporate interactive digital platforms allowing real-time participant queries and feedback. Precision-level consent approaches further stratify information delivery based on participants’ digital literacy and risk exposure.

Such models enable continuous transparency regarding algorithm updates while preserving participant autonomy and ethical compliance throughout the trial lifecycle.

### 6.2. Adopting Standardized Reporting Frameworks

Standardized reporting is necessary to ensure transparency, reproducibility, and comparable results for clinical trials involving AI. The SPIRIT-AI (Standard Protocol Items: Recommendations for Interventional Trials–Artificial Intelligence) extension [9] offers very specific guidelines concerning the inclusion of AI-specific considerations in trial protocols, for example, considerations for algorithm versioning, data handling, and monitoring for performance drift between different sites. In a similar fashion, CONSORT-AI elaborates on the Consolidated Standards of Reporting Trials to accommodate AI-specific requirements on trial results so that fundamental information must be clearly explained, such as the flow of participants, algorithm results, and interpretability [24].

The MI-CLAIM (Minimum Information About Clinical Artificial Intelligence Modeling) checklist [12] complements these standards, emphasizing the importance of model documentation before deployment, the provenance of data, and strategies to monitor after deployment. With the adoption of these standards, in addition to ensuring greater scientific rigor, they will be instrumental in guiding regulatory review and clinical uptake.

### 6.3. Standardizing Endpoints and Comparators

Unlike classical interventions, AI systems may evolve once deployed, thus posing a few challenges for endpoint standardization. The endpoints in an AI trial should be clinically meaningful and reproducible and are directly relevant to patient outcomes [2,3]. The study comparators, whether human experts or standard clinical pathways, need to be duly considered to genuinely reflect clinical practice rather than being subjected to idealized conditions [5] (Table 5).

**Figure 4:**
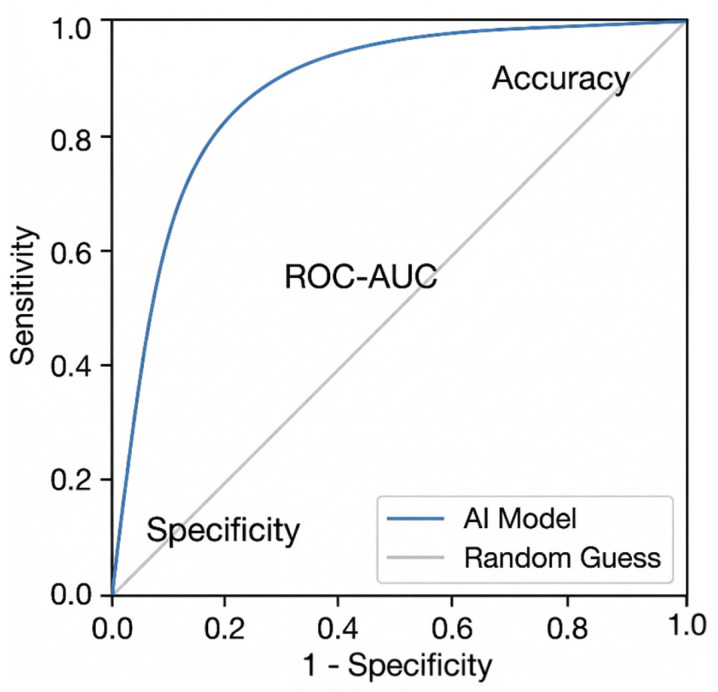
Relationship between Accuracy, Sensitivity, Specificity, and ROC-AUC in AI Clinical Trials, (Figure adapted from [11,16]).

Ref. [25] states that one must consider the clinical domain and the intended use case of the AI system when choosing comparators. For instance, if an AI is in the cardiovascular imaging domain, it should be benchmarked against cardiologists with domain knowledge rather than general radiologists. Such specificity in comparators would ensure a fair and relevant performance evaluation (Figure 4).

## 7. Oncology Supports

### 7.1. Oncology: AI-Assisted Prescreening

**Figure 5:**
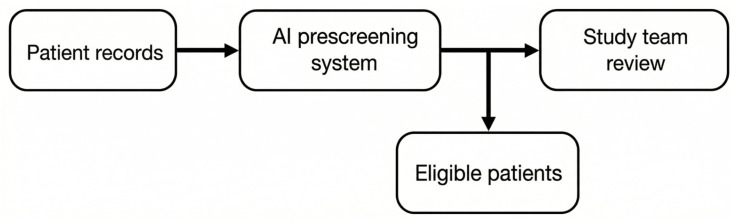
AI-assisted prescreening workflow in oncology trials, adapted from Calaprice-Whitty et al., 2020 [10].

Artificial intelligence offers the greatest potential for participant recruitment and screening in oncology clinical trials. Ref. [10] showed the inequality in prescreening by AI in comparison to the standard way of recruiting in three oncology trials. The potential dynamics are critically important in oncology research, where rapid recruitment is essential for the trial of a novel intervention. The typical AI tool sifted through the vast electronic health record database to make either manual interventions or no manual interventions in matching patient eligibility criteria to increase recruitment throughput. Figure 5 displays the working scheme of AI-assisted prescreening in oncology trials, showing its role in automating eligibility checks and streamlining communication with clinical sites. These types of enhancements will enable novel AI medical devices to be brought into trials with decreased administrative burden and greater patient matching accuracy.

### 7.2. Cardiovascular: Lessons from Oncology Trial Designs

Although cardiovascular treatment trials with AI are still developing, trial methodologies, adapting those of oncology, provide a very important interface for the integration of AI in this field. Ref. [25] argues from the viewpoint of AI-powered recruitment, adaptive endpoints, and biomarker integration implemented in oncology research, attempting to apply this knowledge to cardiovascular research. The authors stress that AI could be used for stratification of cardiovascular trial participants, selecting endpoints through continuous learning, and implementing adaptive trial designs that economize on work intensity without compromising validity. Such adaptive designs would be quite important when testing AI as a medical device in cardiovascular diagnostics, given the peculiarities posed by patient heterogeneity and data collection that spans many years, thereby providing unique challenges. Drawing lessons from the well-developed AI market trial structures of oncology gives cardiovascular research a pathway to speeding up regulatory approval without compromising the dignity of safety and effectiveness evaluation.

### 7.3. Dermatology: Skin Cancer AI Teledermatology Trial

**Figure 6:**
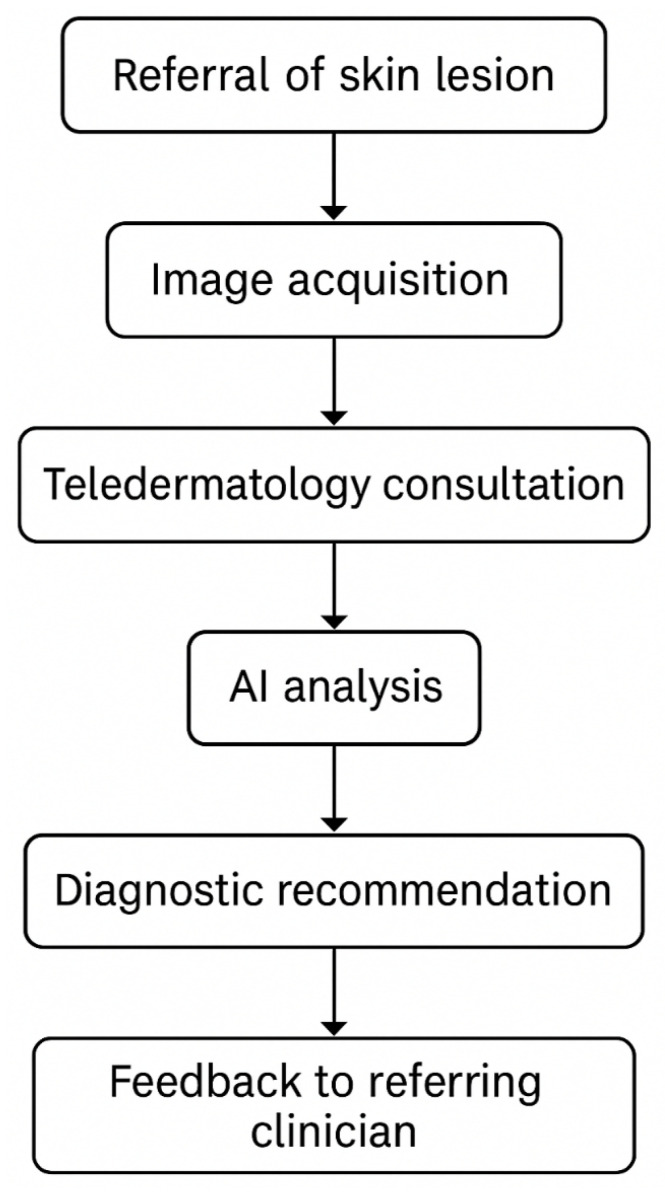
AI integration in skin cancer teledermatology services, adapted from Marsden et al., 2024 [11].

In dermatology, AI has also been evaluated as a diagnostic entity for skin cancer detection through teledermatology services. A UK-based clinical trial of the AI system in a skin-cancer-teledermatology workflow has been reported by [11]. High sensitivity and specificity were demonstrated by the trial in identifying malignant lesions, thereby showing AI’s capability to assist dermatologists in diagnosis in a remote context. Importantly, operationalization in real conditions, with variable image quality and asynchronous consultations, is used for a promising pragmatic evaluation of the AI device. Figure 6 shows the conceptual integration of AI into teledermatology services, showing patient-to-clinician workflows and decision support touch-points. This system aims to serve scalable, accessible diagnostic support tools, especially to a healthcare system in need of resources.

## 8. Regulatory Requirements

### 8.1. Comparison of Trial Approaches Across Regions

The administration and organization of clinical trials for AI as a medical device (AIaMD) are vastly different from country to country due to disparities in regulatory traditions, healthcare structures, and market needs. For instance, in the United States, the FDA has set forth some adaptive regulatory pathways for AI/ML-based devices like the proposed Predetermined Change Control Plan, allowing iterative improvements without requiring a new submission every single time [26]. On the other side of the Atlantic, the EMA operates under the larger umbrella of the EU Medical Device Regulation, which [27] notes has bolstered safety and performance criteria but has not been as accommodating to solutions based on continuous learning algorithms. Another example is that Japan’s PMDA places tremendous emphasis on structured post-market surveillance, as has been described by [7]. Watching AI’s work in the real world is very important. The MHRA in the UK and CDSCO in India are also evolving, with the latter putting some AI-specific considerations into the device classification and approval processes [26]. Such divergences thus create both opportunities for mutual learning and challenges for the coordination of multinational trials.

### 8.2. Strengths and Weaknesses of Current Regulatory Models

Although regulatory agencies share the common objective of ensuring safety and effectiveness, their approaches to AIaMD oversight differ substantially in philosophy, maturity, and operational flexibility. These differences create both opportunities for innovation and barriers to global consistency.

The FDA framework is comparatively proactive and innovation-oriented, incorporating mechanisms such as predetermined change control plans and lifecycle monitoring that explicitly acknowledge the adaptive nature of AI systems. This approach enables iterative improvement without repeated full submissions, thereby supporting rapid technological evolution. However, critics argue that increased flexibility may shift greater responsibility to post-market controls, potentially increasing reliance on real-world surveillance rather than robust pre-market evidence.

In contrast, the European Union Medical Device Regulation (MDR) emphasizes precaution and formal conformity assessment. While this model strengthens traceability, documentation, and patient safety safeguards, its comparatively rigid structure may be less well aligned with continuously learning algorithms that require frequent updates. Several authors note that this conservatism may delay deployment or discourage adaptive innovation.

Emerging jurisdictions, including India and other lower-resource settings, often face additional implementation challenges, including limited post-market infrastructure and evolving regulatory capacity. As a result, regulatory expectations may be less clearly defined, creating uncertainty for developers and healthcare providers.

Taken together, these models reflect an unresolved tension between two priorities:

(i) safeguarding patients through stringent pre-market evidence, and
(ii) enabling iterative innovation through lifecycle regulation.

Current frameworks tend to favor one objective over the other rather than integrating both. This imbalance highlights the need for hybrid approaches that combine structured pre-market validation with systematic post-deployment monitoring.

#### Limitations and Risk Events in AIaMD Clinical Trials

While many reported AIaMD trials demonstrate favorable outcomes, several studies document trial limitations and failure scenarios. These include premature trial termination due to algorithmic performance drift, reduced generalizability caused by dataset bias, and regulatory delays arising from insufficient post-market monitoring plans.

Analysis of such cases highlights the necessity of predefined change control plans, continuous performance monitoring, and bias surveillance as integral components of AIaMD trial design.

### 8.3. Opportunities for Global Harmonization

The global landscape of regulation of AIaMDs is at a stage where harmonization would greatly benefit manufacturers, regulators, and patients alike. The harmonization could target the standardization of the definitions, evidence requirements, and performance standards, as given in the MI-CLAIM checklist by [12] and the SPIRIT-AI protocol guidelines by [9]. There would be less duplication of effort when requisites for the baseline are common in multinational trials, leading to quicker approvals and comparisons of performance in the market later. Ref. [24] cites the example of how standardized reporting guidelines in certain domains, such as gastrointestinal endoscopy, can provide a model for wider implementation. The WHO could also play an important role in coordinating the development of principles for global AIaMD trials, ensuring that innovations can be accessible to low-resource settings without compromising safety and efficacy standards.

### 8.4. The Role of Industry–Academia Partnerships

Such collaborations are especially necessary to promote AIaMD evaluation and adoption and thereby bridge the gap between technical innovation and clinical validation. Ref. [28] has described instances where multidisciplinary workshops, such as those convened by NIH/RSNA/ACR, were utilized to bring together stakeholders from engineering, medicine, and regulatory science for defining translational research priorities. These collaborations can also foster the creation of diverse datasets of the highest quality that adhere to regulatory guidelines concerning representativeness and bias mitigation, as discussed in [22]. Additionally, there is an inverse proclivity of academic setups to provide methodological skills for the establishment of a rigorous trial, whereas industry partners can supply the necessary resources and opportunities for implementation. Ref. [14] argues that when research is aligned with regulators and policy agencies, AIaMD innovations will be relevant to actual life; in contrast, relevance will be mainly technically oriented and less ethereal. Increasing such collaborations internationally will enable harmonized trial designs and enhanced regulatory review efficiency across various jurisdictions.

## 9. Future Trends

### 9.1. Key Messages and Future Priorities

AIaMD possesses promise to many health branches, from oncology to cardiovascular medicine and dermatology. Yet, efficiency development and deployment are impeded by the diversity of trial methodologies and regulatory pathways. Rivera et al. [9] accentuated that applying a formal reporting framework, e.g., SPIRIT-AI, across the board is a must for transparency, ensuring reproducibility, and facilitating comparability across studies. Kiseleva [1], on the other hand, stresses the urgency of harmonizing general AI regulation with that concerning medical devices so that they do not become duplicative but rather complement one another and take into consideration unique algorithmic risks. Without a cohesive and predictable regulatory ecosystem, physicians are bound to face clinical stagnation with innovative AI devices.

Importantly, expectations for AIaMD evaluation have evolved considerably over the past decade. Earlier publications (2017–2019) largely focused on demonstrating diagnostic accuracy and feasibility within controlled datasets. Over time, emphasis has shifted toward broader considerations, including reporting transparency, dataset representativeness, bias mitigation, and lifecycle governance. More recent guidance increasingly recognizes that performance alone is insufficient without evidence of workflow integration, human oversight, and sustained post-market monitoring. This progression reflects a maturation of the field—from proof-of-concept validation toward implementation science and real-world accountability—underscoring the need for trial designs that capture both technical and operational performance

### 9.2. Policy Implications

The global harmonization efforts continue to be scattered, and various jurisdictions, such as the FDA, EMA, MHRA, and CDSCO, impose varied demands with respect to clinical validation and post-market surveillance. Any framework alignment, according to Chauhan et al. [26], would only fast-track regulatory review and allow sponsors to conduct multinational trials, thereby generating more compelling evidence. Ref. [7] argues that post-marketing surveillance strategies must be integrated to make sure real-world performance is observed and issues are detected that might not arise in a controlled setting. Given that AI is adaptive, such policies must also consider continuous learning systems as a safeguard to ensure that software updates have not unintentionally compromised safety or efficacy.

### 9.3. Call for Coordinated International Guidelines

The need for coordinated, evidence-based, and globally recognized AIaMD trial standards is urgent. Niemiec [27] argues that current device regulations, while necessary, are insufficiently tailored to the dynamic and data-driven characteristics of AI. Collaborative models between regulators, industry, and academia, as proposed by Allen et al. [28], offer a pathway to consensus, leveraging multidisciplinary expertise to refine trial designs and validation metrics. In moving forward, a unified framework, built on transparency, post-market vigilance, and cross-border cooperation, will be vital to ensuring AI medical devices are safe, effective, and equitably accessible worldwide.

### 9.4. Perspectives for Clinical and Assistive Practice

From a clinical and assistive practice perspective, AIaMD adoption is better conceptualized as a pathway-level implementation challenge rather than solely a technical performance problem. Successful deployment depends not only on algorithm accuracy but also on workflow compatibility, human oversight, training, and governance arrangements that clarify how AI outputs are interpreted and acted upon [5,9].

Accordingly, evidence generation should consider implementation outcomes such as workflow disruption, clinician oversight burden, escalation logic, and patient acceptability alongside conventional diagnostic endpoints. Trials that evaluate only technical performance may demonstrate limited generalizability to routine care settings [6,9].

Clear delineation of accountability is also necessary. When AI outputs inform triage or treatment decisions, responsibility for review and final judgment should remain explicit to reduce automation bias and safety risks [5,13]. Governance models that define escalation procedures and documentation standards may further support safe integration.

Sustained post-deployment monitoring represents another critical consideration. Model performance may change over time due to evolving patient populations, new devices, or clinical practices, necessitating ongoing surveillance for drift and revalidation [7,12]. Real-world evidence approaches have been proposed to capture performance trends and disparities that may not be evident in controlled trials [15].

Finally, equitable adoption requires systematic assessment of subgroup performance and bias mitigation strategies. Stratified evaluation across demographic groups is increasingly recommended to ensure that AI systems do not exacerbate health disparities [13,14].

#### Priority Roadmap for AIaMD Clinical Trial Harmonization

To translate harmonization principles into practice, implementation mechanisms must be clearly defined.

Short-term (1–2 years):

Adoption of standardized reporting frameworks such as SPIRIT-AI, CONSORT-AI, and MI-CLAIM across journals, sponsors, and regulators to improve transparency, reproducibility, and comparability of evidence.

Medium-term (3–5 years):

Formation of coordinated working groups involving regulators, industry, and academic stakeholders—potentially aligned with existing international collaboration models (e.g., WHO technical committees or ICH-style expert working groups)—to establish shared expectations for adaptive validation, change control planning, and real-world evidence monitoring.

Long-term (5–10 years):

Development of internationally endorsed guidance outlining minimum evidence requirements, lifecycle management standards, and post-market surveillance frameworks for AIaMD. Such consensus standards would facilitate mutual recognition across jurisdictions and reduce duplication of regulatory submissions, particularly for multinational trials.

By anchoring harmonization efforts within established global governance structures rather than ad hoc initiatives, these steps may enable consistent oversight while preserving regional flexibility.

## Figures and Tables

**Table 1:** Comparative trial phases for traditional devices vs. AIaMD.

Phase	Traditional Medical Devices	AI as a Medical Device (AIaMD)
Preclinical/Feasibility	Bench testing, animal studies, and early feasibility studies	Data set curation and annotation, model training, and initial internal validation
Pivotal/validation	Large-scale RCTs, fixed design, pre-specified endpoints	Adaptive trials, interim analyses, and continuous learning systems
Post-Market Surveillance	Periodic safety updates, adverse event reporting	Real-world performance monitoring, algorithm updates, bias surveillance

**Table 2:** Summarizes the benefits and limitations of simulation-based testing for AIaMD.

Benefit	Limitation
Rapid scalability to multiple scenarios	May not fully replicate real-world variability
No patient safety risk	Risk of overfitting to synthetic data
Enables early bias detection	Requires gigh quality virtual modeling data

**Table 3:** Key Quality Attributes for AIaMD Datasets (adapted from Wang et al., 2019 [22]; Challen et al., 2019 [13]; Dwivedi et al., 2019 [14]).

Attribute	Description	Importance for AIaMD
Coverage	Representation of all relevant patient demographics, clinical conditions, and device use cases.	Ensures AI performance across diverse populations and reduces bias.
Annotation Standards	Use of standardized, validated labeling protocols and domain expert review	Improves model accuracy and reproducibility of results
Metadata Completeness	Inclusion of acquisition parameters, device settings, environmental conditions, and patient characteristics	Supports traceability, model interpretability, and compliance with regulatory documentation
Data Quality Control	Processes to detect and correct errors, remove duplicates, and manage missing values	Maintains data integrity and prevents degradation of AI model performance
Privacy Compliance	Conformance with HIPAA, GDPR, and other relevant data protection laws	Protects patient rights and ensures regulatory approval

**Table 4:** Ethical and Legal Considerations in AI Clinical Trials.

Ethical/Legal Domain	Key Challenges	References
Informed consent	Complexity of AI explanations; dynamic consent needs	Youssef et al., 2024 [6]; Dwivedi et al., 2019 [14]
Transparency and Accountability	Black box algorithms; unclear responsibility for errors	Rivera et al., 2020 [9]; Challen et al., 2019 [13]
Explainability	Clinician and patient trust; understanding probabilistic outputs	Havey and Oakden–Rayner, 2020 [5]
Patient Safety	Algorithmic drift; cybersecurity threats	Youssef et al., 2024 [6], Gomase et al., 2025 [18]

**Table 5:** Key reporting standards and their focus areas.

Standard/Checklist	Focus Area	Example Application	Reference
SPIRIT-AI	Protocol design for AI trials	Inclusion of AI-specific risk monitoring	Rivera et al., 2020 [9]
CONSORT-AI	Trial reporting standards	Clear reporting of AI decision outputs	Bilal et al., 2020 [24]
MI-CLAIM	AI model documentation	Pre- and post- deployment model details	Norgeot et al., 2020 [12]

## Data Availability

No new data were created or analyzed in this study.

## Supplementary Materials

The following supporting information can be downloaded at: https://www.mdpi.com/article/10.3390/jcm15051937/s1, S1—Full search strategy (PubMed); S2—PRISMA-ScR checklist; S3—PRISMA-ScR flow diagram. (The PRISMA-ScR flow diagram is also presented in the main text as Figure 1).
